# Cortical thinning and functional reorganization as well as related transcriptomic signatures during early motor recovery following acute basal ganglia ischemic stroke

**DOI:** 10.1186/s12984-026-02207-z

**Published:** 2026-11-03

**Authors:** Huachun Huang, Xueqin Jiang, Yang Liu, Shunzu Lu, Hongyu Lin, Yanyan Liu, Zhijian Liang

**Affiliations:** 1 Department of Neurology, The First Affiliated Hospital of Guangxi Medical University, Nanning, China; 2 Department of Radiology, The First Affiliated Hospital of Guangxi Medical University, Nanning, China

**Keywords:** Acute basal ganglia ischemic stroke, Cortical thickness, Functional connectivity, Machine learning, Allen Human Brain Atlas

## Abstract

**Background:**

Acute basal ganglia ischemic stroke (ABGIS) commonly causes severe motor impairment, yet early spatiotemporal patterns of cortical morphological alterations and functional reorganization alongside the spatially coupled transcriptomic signatures remain unclear.

**Methods:**

Twenty-six ABGIS patients and thirty-three normal controls (NCs) received multimodal MRI and neurological examinations, and patients were reassessed at 1-month follow-up. Cortical morphological changes and brain functional reorganization were analyzed based on cortical thickness (CT) and functional connectivity (FC). We identified the most discriminative FC features based on machine learning (ML) classification and interpretability analyses, and investigated transcriptomic signatures associated with FC alterations via the Allen Human Brain Atlas.

**Results:**

No differences in CT were found between baseline ABGIS patients (ABGIS_1) and NCs. Compared with ABGIS_1, follow-up patients (ABGIS_2) exhibited reduced CT in the left postcentral gyrus (PoCG.L), left superior parietal lobule (SPL.L), and left supramarginal gyrus (SMG.L). Compared with NCs, ABGIS_1 showed decreased FC from PoCG.L/SPL.L to sensorimotor cortex and occipital cortex. ABGIS_2 presented elevated FC relative to ABGIS_1 between PoCG.L and the right precentral gyrus (PreCG.R), and between SPL.L and the bilateral precentral gyrus as well as right superior occipital gyrus. Longitudinal increases in FC between PoCG.L/SPL.L and PreCG.R were positively correlated with improvements in Fugl-Meyer Assessment scores. ML models coupled with interpretability analyses preliminarily demonstrated that FC between PoCG.L/SPL.L and PreCG.R made prominent contributions to classification. Transcriptomic analysis revealed that gene sets correlated with FC alterations were mainly enriched in neuronal synaptic structure and synaptic signaling, chromatin regulation, as well as DNA and RNA metabolism.

**Conclusions:**

This study verifies progressive cortical thinning and adaptive functional reorganization during early motor recovery following ABGIS, with such brain functional alterations paralleling distinct transcriptomic signatures, providing neuropathological and spatial transcriptomic clues for interpreting early motor recovery.

**Supplementary Information:**

The online version contains supplementary material available at https://doi.org/10.1186/s12984-026-02207-z.

## Introduction

As the most common subtype of stroke, ischemic stroke is a leading cause of death and disability worldwide [1]. The basal ganglia are a cluster of subcortical nuclei responsible for motor control, motor learning, and executive function [2]. Due to inadequate vascular anastomosis, this region is particularly susceptible to ischemic injury [3]. Consequently, ischemic injury in this region typically leads to acute basal ganglia ischemic stroke (ABGIS), with subsequent motor impairment of varying severity.

Surface-based morphometry (SBM) and voxel-based morphometry (VBM) are two widely utilized approaches for assessing cortical morphology in structural MRI [4]. Accumulating evidence suggests that cortical thickness (CT) derived from SBM might be more appropriate than gray matter volume (GMV) measured by VBM for evaluating morphological abnormalities in brain regions [4, 5]. As a common metric derived from resting-state functional MRI (rs-fMRI), functional connectivity (FC) reflects the coordinated activity between different brain regions based on the temporal similarity of blood oxygen level-dependent (BOLD) signals [6]. The combined analysis of CT and FC enables a more comprehensive characterization of post-stroke brain injury and repair. Notably, neurological recovery after stroke follows a distinct time course. Evidence from animal models demonstrates that the period of peak neurological recovery occurs within the first month after stroke [7]. Accordingly, exploring early cortical morphological changes and functional reorganization after stroke is of great significance for providing insights into post-stroke motor recovery.

Although numerous multimodal MRI studies have demonstrated structural and functional brain alterations in stroke patients, identifying pivotal and interpretable imaging phenotypes remains clinically critical. The rapid development of machine learning (ML) in neuroimaging provides new directions for the early detection and prediction of brain diseases, while also offering novel insights into the discovery of sensitive imaging phenotypes [8]. Classification models based on ML facilitate the evaluation of the discriminative power of various MRI features in distinguishing stroke patients from healthy controls, thereby advancing clinical detection and post-stroke functional assessment [9, 10]. However, conventional ML models often suffer from limited interpretability, making it difficult to trace the contribution of individual features to their decisions. To address this limitation, SHapley Additive exPlanation (SHAP) has been increasingly applied to quantify the contribution of each imaging feature, thus improving the biological plausibility and clinical value of model outputs [11].

Despite extensive investigations into structural and functional brain alterations using multimodal MRI and ML, the spatial transcriptomic links to neuroimaging phenotypes in stroke patients remain poorly characterized. To bridge this gap, the microarray dataset from the Allen Human Brain Atlas (AHBA) has been widely employed to identify transcriptomic profiles linked to human neuroimaging features [12]. As an effective method for such analyses, partial least squares (PLS) regression enables robust integration between macroscopic neuroimaging and microscopic transcriptomics across brain regions [13, 14]. Notably, PLS analysis has been shown to characterize transcriptional signatures associated with neuroimaging changes across neuropsychiatric disorders [15, 16]. However, whether it can be applied to identify spatial transcriptomic correlates of early neurological recovery in acute ischemic stroke remains to be explored.

To fill this critical knowledge gap, the present study utilized longitudinal multimodal MRI integrated with ML and spatial neuroimaging-transcriptome association analysis. This study sought to achieve three primary aims: (1) to explore the dynamic alterations in CT and FC linked to motor function across the acute to early recovery phase in patients with ABGIS; (2) to determine the most distinguishing FC features based on ML classification coupled with SHAP analysis; and (3) to investigate the spatial association between FC changes and gene expression profiles based on AHBA transcriptomic data, thereby mapping spatial transcriptomic correlates of early motor recovery.

## Materials and methods

### Participants

A total of 26 patients (23 males, 3 females) diagnosed with ABGIS were enrolled from the First Affiliated Hospital of Guangxi Medical University. All eligible patients met the following criteria: (1) the time interval from symptom onset to MRI examination was within 1 week; (2) MRI confirmed a solitary first-ever ischemic stroke involving the unilateral basal ganglia region; (3) participants were aged 18 years or older; (4) patients were right-handed prior to stroke onset; (5) patients were conscious and able to complete MRI scanning and scale assessments. The exclusion criteria for patients were: (1) a history of other neuropsychiatric disorders or systemic illnesses; (2) severe white matter lesions detected on MRI; (3) failure to complete the 1-month follow-up examination; (4) poor MRI image quality, with head motion greater than 3° or 3 mm observed during image preprocessing. In addition, a total of 33 volunteers (23 males, 10 females) without any history of neuropsychiatric or chronic diseases were recruited as the normal controls (NCs). The NCs were matched with the ABGIS group in terms of gender, age and education level. The interscan interval between baseline (ABGIS_1) and follow-up (ABGIS_2) was 1 month. This study was approved by the Ethics Committee of the First Affiliated Hospital of Guangxi Medical University, and written informed consent was acquired from all participants prior to enrollment.

### Neurological assessment

The severity of neurological deficits in patients with ABGIS was evaluated using the National Institutes of Health Stroke Scale (NIHSS), and motor function was assessed via the Fugl-Meyer Assessment (FMA).

### MRI data acquisition

Imaging data were acquired using a 3.0 T Prisma MRI scanner (Siemens, Germany). Subjects lay supine with the head stabilized by two foam pads and wore earplugs to reduce scanner noise. During MRI scanning, all participants were instructed to stay awake and relaxed, keep their eyes closed, and refrain from active thinking. High-resolution T1-weighted images (T1WI) were acquired for structural analysis with the following parameters: echo time (TE) = 2.98 ms, repetition time (TR) = 2300 ms, flip angle (FA) = 90°, field of view (FOV) = 256 mm × 256 mm, voxel size = 1 mm × 1 mm × 1 mm, slice thickness = 1 mm, 176 slices, and a total scan duration of 5 min and 21 s. Rs-fMRI sequences were acquired for functional analysis using TE = 30 ms, TR = 2170 ms, FA = 90°, FOV = 192 mm × 192 mm, voxel size = 2 mm × 2 mm × 2 mm, slice thickness = 2 mm, 70 slices, and a total scan duration of 6 min and 43 s.

### T1WI data preprocessing and CT analysis

The SPM25 (http://www.fil.ion.ucl.ac.uk/spm) and CAT12 (http://www.neuro.uni-jena.de/cat) were used for T1WI data preprocessing and CT analysis. For patients with right-sided lesions, images were flipped across the midsagittal plane to standardize the left hemisphere as the lesioned side for all patients [17–19]. All images were automatically segmented into gray matter (GM), white matter (WM), and cerebrospinal fluid (CSF), followed by affine registration to the MNI template space and subsequent nonlinear deformation. The projection-based thickness approach was applied to measure cortical thickness and reconstruct the central surface for the left and right hemispheres. The generated CT images were resampled to a 2 mm spatial resolution and smoothed using a Gaussian kernel with 12 mm full-width at half-maximum (FWHM).

### Rs-fMRI data preprocessing and FC analysis

RESTplus V1.31 (http://restfmri.net/forum/restplus) was used for rs-fMRI data preprocessing and FC analysis. Consistent with structural images, rs-fMRI images were also flipped across the midsagittal plane for patients with right-sided lesions [17–19]. The preprocessing pipeline comprised the following steps: discarding the initial 10 volumes; performing slice-timing correction; conducting head motion correction; normalizing functional images to MNI space using the DARTEL algorithm; applying a 6 mm FWHM Gaussian kernel for spatial smoothing; detrending linear signals; applying temporal band-pass filtering (0.01–0.08 Hz); and regressing out nuisance signals including 24 motion parameters, WM and CSF signals. Seed-based FC analysis was performed using 6-mm-radius spheres centered at the peak coordinate for brain regions showing significant between-group differences in CT [20]. Each sphere was intersected with the standard gray matter mask. Only voxels falling within both the sphere and the gray matter mask were retained for subsequent seed-based FC analysis. Pearson’s correlation was then analyzed between the average time course of each seed region and the rest of the brain. Subsequently, the obtained correlation coefficients were normalized to z-values via Fisher’s r-to-z conversion to generate individual voxel-wise FC maps for each participant. Following group-level statistical analyses, significant clusters with intergroup FC differences were identified. Mean Fisher-z FC values across all voxels within each significant cluster were then extracted from each individual FC map for subsequent analyses.

### Machine learning analysis

We constructed independent binary classification models for multiple seed-based FC phenotypes to distinguish ABGIS_1 from NCs. For each phenotype, FC features were extracted across all brain regions defined by the Anatomical Automatic Labeling (AAL) atlas and served as inputs to construct separate support vector machine (SVM) models using the Scikit-learn toolkit in Python (version 3.11.9). To ensure robust model evaluation and avoid overfitting, a nested cross-validation (CV) structure was applied [21], consisting of an outer 10-fold stratified CV repeated 10 times and an inner 5-fold CV for hyperparameter tuning via grid search. Hyperparameter tuning was restricted to training data within each inner fold. Feature standardization was implemented using StandardScaler. While scaler fitting for inner fold parameter evaluation was performed on inner training subsets, the final scaler used for outer sample prediction was refitted on the complete training subset of each outer fold. No preprocessing was carried out on the full dataset of the study cohort. Model performance was evaluated using the area under the receiver operating characteristic curve (AUC), accuracy, sensitivity, and specificity. To quantify the uncertainty of out-of-sample performance, we additionally computed 95% confidence intervals (CIs) for AUC using bootstrap resampling. A fully nested permutation test involving 1000 random label shuffles was conducted to assess statistical significance at the model level. Each label permutation repeated the full nested CV workflow from scratch, with no reuse of pretrained models or precomputed outputs obtained under real label conditions. SHAP analysis was performed to quantify the contributions of each feature to model predictions [22]. Notably, no additional iterative feature elimination was conducted within any CV fold across the entire modeling pipeline.

### Gene expression data preprocessing

Gene expression data were obtained from the AHBA dataset [23] (https://human.brain-map.org). This dataset contains expression profiles of over 20,000 genes across 3,702 independent tissue samples from six postmortem adult donors (five males and one female, aged 24–57 years). Since only two donors in the AHBA provided right hemisphere data, this study exclusively examined the left hemisphere [14]. Standardized preprocessing workflows for AHBA microarray data were carried out with the abagen toolbox [24] (https://github.com/rmarkello/abagen). The detailed preprocessing steps were as follows: probe-to-gene re-annotation with exclusion of probes lacking unambiguous gene mapping or corresponding valid Entrez gene identifiers; intensity-based probe filtering to remove probes with signals not exceeding background noise in at least 50% of samples; selection of the probe with the highest correlation to RNA-seq data for each gene; assignment of tissue samples from the left hemisphere to the nearest AAL atlas parcel within a 2 mm Euclidean distance threshold, and exclusion of tissue samples falling outside this distance threshold; normalization of expression values within each donor only for tissue samples assigned to atlas parcels using scaled robust sigmoid transformation [14]. Accordingly, this procedure yielded a final transcriptional matrix of 54 regions × 15,633 genes.

### Neuroimaging-transcriptome spatial association analysis

We generated cross-sectional and longitudinal maps of FC changes across the whole brain (t-maps) based on different FC phenotypes. PLS regression was applied to assess spatial associations between the t-maps and gene expression profiles from the AHBA dataset. Two distinct PLS regression models were constructed. In one model, t-statistic values from the comparison of ABGIS_1 versus NCs served as the response variable. In the other model, paired t-statistic values derived from ABGIS_2 versus ABGIS_1 were treated as the response variable. For both models, the predictor variables were taken from the same matrix of gene expression. Both the predictor and response variables were standardized using z-score transformation. PLS components were derived from linear combinations of gene expression values. The first component (PLS1) was commonly selected to best characterize the covariance patterns of high-dimensional data matrices [25]. The statistical significance of PLS results was assessed using spherical permutation testing with 10,000 iterations to account for spatial autocorrelation [15]. Subsequently, bootstrap resampling with 1000 iterations was performed to estimate the stability of gene weights in PLS1 [15]. The Z-score of each gene was calculated as its PLS1 weight divided by the bootstrap standard error, and all genes were ranked by Z-score. Genes with a Z-score > 3 and false discovery rate (FDR)-corrected *P <* 0.05 were regarded as PLS1 + genes, whereas those with a Z-score < -3 and FDR-corrected *P <* 0.05 were regarded as PLS1- genes. We then performed overlap analyses independently for the PLS1 + and PLS1- genes derived from PLS regression models across different FC phenotypes. The overlapping gene sets for PLS1 + and PLS1- were retained separately for enrichment analyses.

### Enrichment analysis

Using the overlapping PLS1 + and PLS1- gene sets, we separately performed functional enrichment analyses of Gene Ontology (including biological processes, cellular components, and molecular functions) and Kyoto Encyclopedia of Genes and Genomes pathways via the Metascape platform (https://metascape.org). Notably, the 15,633 genes retained after AHBA preprocessing were set as the custom background gene set for enrichment analysis. The threshold for all obtained pathways was set at an FDR-corrected *P* < 0.05.

### Statistical analysis

Statistical analysis of demographic and clinical data was performed using SPSS 26.0. Normally distributed continuous variables were reported as mean ± standard deviation, whereas those with non-normal distribution were described as median (25th percentile, 75th percentile). The two-sample t-test was performed to analyze differences in normally distributed quantitative data, while the Mann-Whitney U test was applied to non-normally distributed quantitative data. In addition, the chi-square test was used to assess intergroup differences in qualitative data. Between-group differences in CT for both ABGIS_1 versus NCs and ABGIS_2 versus NCs were examined using two-sample t-tests with age, sex, and education as covariates. Similarly, between‑group differences in FC for both ABGIS_1 versus NCs and ABGIS_2 versus NCs were assessed with age, sex, education, and mean framewise displacement (FD) as covariates. Paired t-tests were performed separately to evaluate changes in CT or FC from ABGIS_1 to ABGIS_2. For brain imaging analyses, statistical significance was defined as a voxel-level uncorrected *P* value < 0.001 and a cluster-level family-wise error (FWE)-corrected *P* value < 0.05 (two-tailed). Furthermore, correlation analyses were conducted to evaluate the relationships between changes in CT/FC and alterations in NIHSS/FMA scores. Changes in CT/FC (△CT/△FC) and NIHSS/FMA scores (△NIHSS/△FMA) were calculated as the value at follow-up (ABGIS_2) minus the value at baseline (ABGIS_1). The statistical threshold for correlation analyses was set at *P* < 0.05/14 via Bonferroni correction, where 14 represented the total number of correlation analyses.

## Results

### Demographic and clinical characteristics

A total of 26 patients with ABGIS and 33 NCs were ultimately analyzed in this study. Demographic characteristics were comparable between the two groups, with no significant differences observed in age, sex, or education level (all *P* > 0.05). All 26 ABGIS patients completed a one-month follow-up examination. Lesion lateralization was right-sided in 12 patients and left-sided in 14. Longitudinal comparisons revealed that the NIHSS score was significantly lower in ABGIS_2 than in ABGIS_1 (*Z* = -4.342, *P* < 0.001), whereas the FMA score was significantly higher in ABGIS_2 than in ABGIS_1 (*Z* = 4.460, *P* < 0.001). Detailed data are presented in Table 1 and Supplementary Fig. 1.

**Table 1:** Demographic and clinical features of patients with ABGIS and NCs

	Median (P25, P75) / Mean ± SD			Statistical value	*P*-value
	ABGIS_1 (*n* = 26)	ABGIS_2 (*n* = 26)	NCs (*n* = 33)		
Age (years)	54.00 ± 10.25	-	55.30 ± 9.12	-0.516a	0.608
Sex (male/female)	23/3	-	23/10	2.981b	0.084
Education (years)	12.00 (9.00, 12.75)	-	12.00 (9.00, 15.00)	-0.110c	0.912
Stroke hemisphere (right/left)	12/14	-	-	-	-
NIHSS	3.00 (2.00, 4.25)	1.00 (0.00, 2.00)	-	-4.342d	< 0.001
FMA	85.50 (78.75, 90.25)	96.00 (88.75, 99.00)	-	4.460d	< 0.001

Abbreviations: P25, 25th percentile; P75, 75th percentile; SD, standard deviation; ABGIS_1, patients with acute basal ganglia ischemic stroke at baseline; ABGIS_2, patients with acute basal ganglia ischemic stroke at follow-up; NCs, normal controls; NIHSS, National Institutes of Health Stroke Scale; FMA, Fugl-Meyer Assessment

Statistical tests: a Two-sample t-test; ^b^ Chi-square test; ^c^ Mann-Whitney U-test; ^d^ Wilcoxon signed-rank test

*P* < 0.05 was considered statistically significant

### CT analysis

**Fig. 1:**
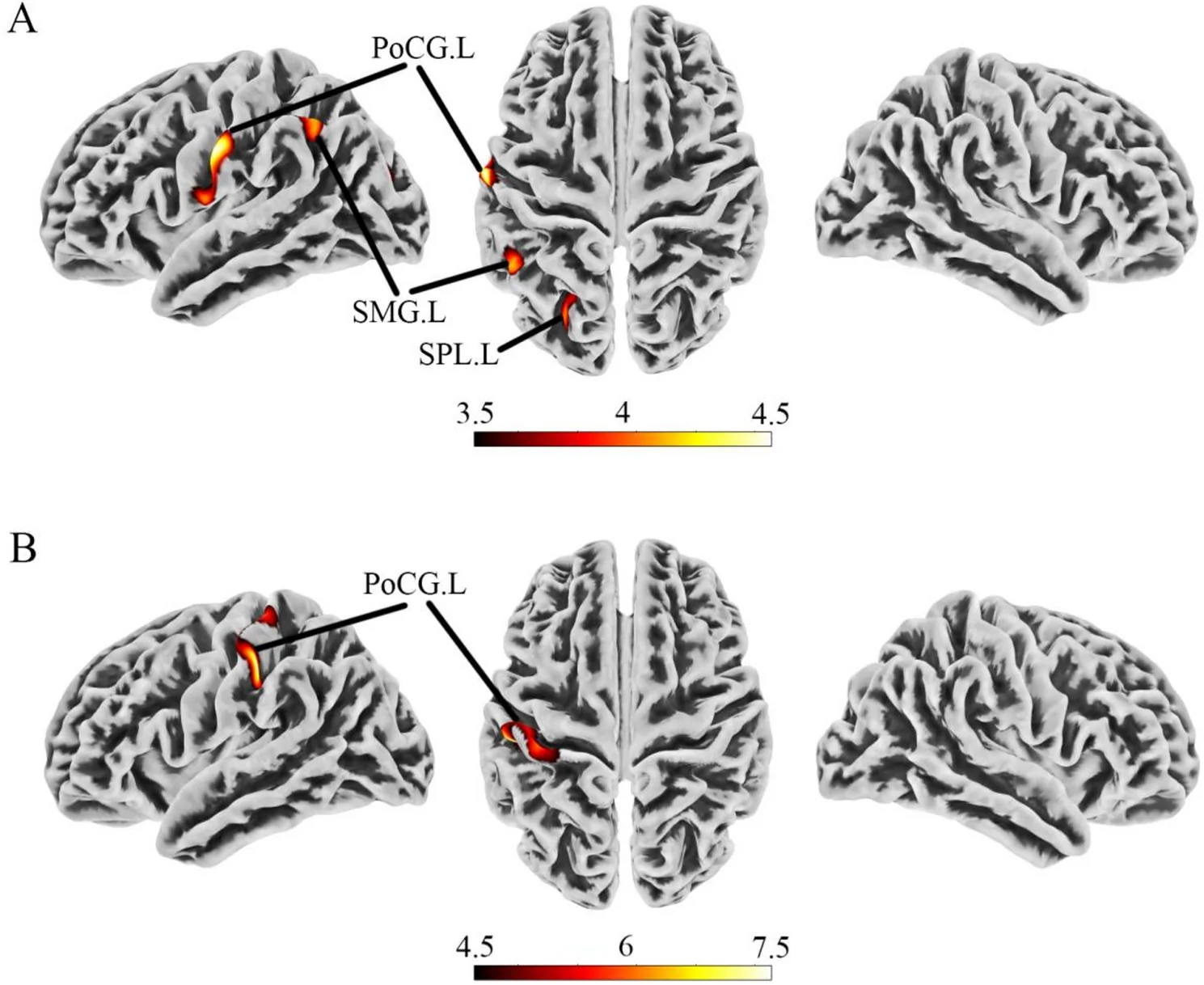
Brain regions with significantly decreased cortical thickness in group comparisons. (**A**) ABGIS_2 versus ABGIS_1. (**B**) ABGIS_2 versus NCs. PoCG.L: left postcentral gyrus; SPL.L: left superior parietal lobule; SMG.L, left supramarginal gyrus

Compared with NCs, no significant differences in CT were detected in ABGIS_1. Relative to ABGIS_1, ABGIS_2 showed significantly reduced CT in the left postcentral gyrus (PoCG.L), left superior parietal lobule (SPL.L), and left supramarginal gyrus (SMG.L) (Table 2; Fig. 1A). Furthermore, ABGIS_2 exhibited significantly decreased CT in PoCG.L compared with NCs (Table 2, Supplementary Table 1, Fig. 1B).

**Table 2:** Brain regions with altered cortical thickness

Brain region	Cluster size	Peak MNI coordinates			T-value
		x	y	z	
ABGIS_2 < ABGIS_1					
PoCG.L	193	-55	-29	41	4.51
SPL.L	172	-16	-74	17	4.14
SMG.L	130	-39	-60	44	4.20
ABGIS_2 < NCs					
PoCG.L	397	-44	-25	36	7.28

Note: Atlas parcellation: Desikan-Killiany atlas. Voxel-level threshold: uncorrected *P* < 0.001; cluster-level threshold: Family-wise error (FWE)-corrected *P* < 0.05 (two-tailed). ABGIS_1, patients with acute basal ganglia ischemic stroke at baseline; ABGIS_2, patients with acute basal ganglia ischemic stroke at follow-up; NCs, normal controls. PoCG.L, left postcentral gyrus; SPL.L, left superior parietal lobule; SMG.L, left supramarginal gyrus

### FC analysis

**Fig. 2:**
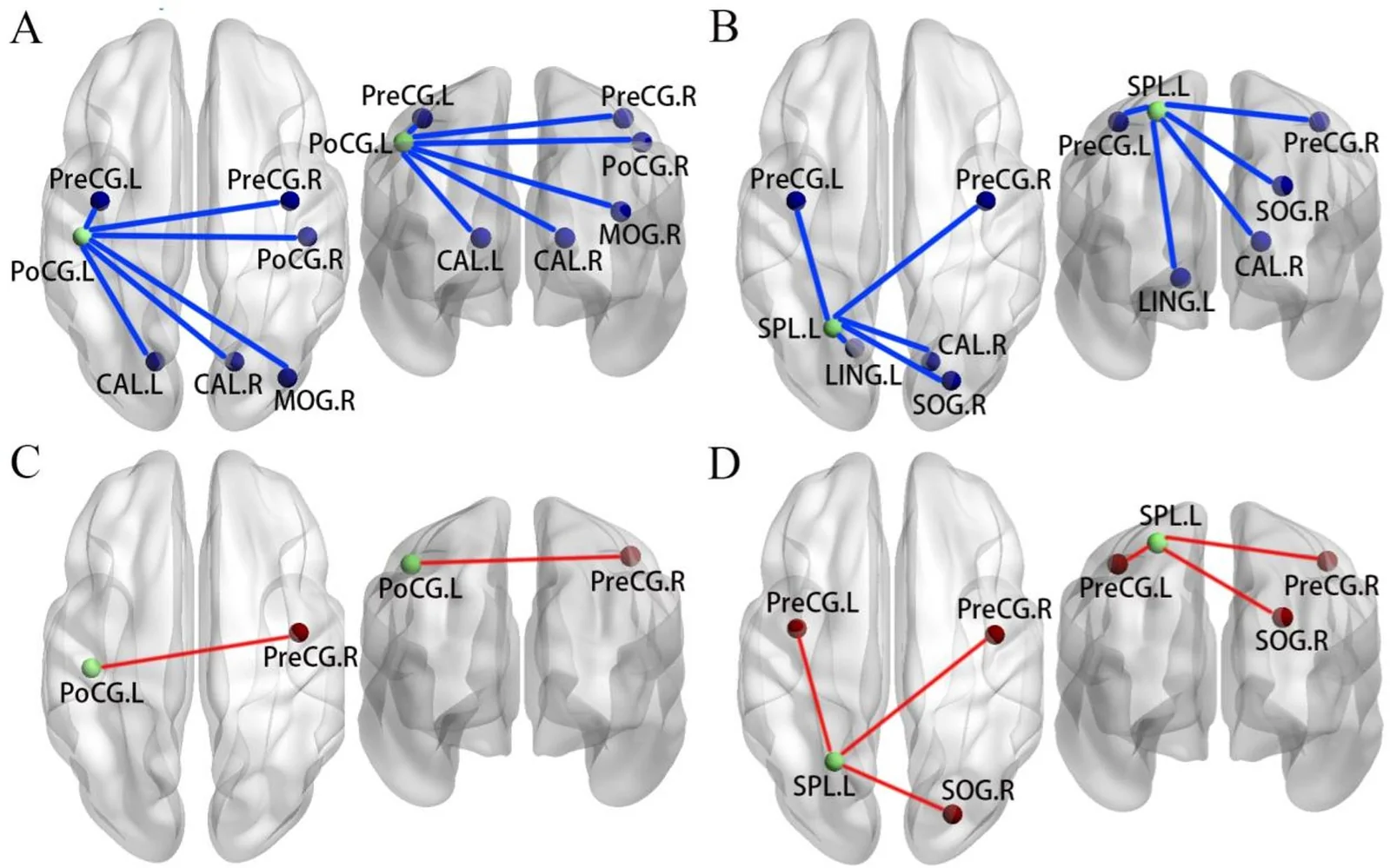
Intergroup functional connectivity differences seeded in the left postcentral gyrus and left superior parietal lobule. (**A**, **B**) Functional connectivity was significantly decreased in ABGIS_1 relative to NCs. (**C**, **D**) Functional connectivity was significantly increased from ABGIS_1 to ABGIS_2. Green nodes denote the seed regions: the left postcentral gyrus (PoCG.L) and left superior parietal lobule (SPL.L). Blue nodes represent target regions with significantly reduced functional connectivity, including the right precentral gyrus (PreCG.R), left precentral gyrus (PreCG.L), right postcentral gyrus (PoCG.R), right middle occipital gyrus (MOG.R), left calcarine cortex (CAL.L), right calcarine cortex (CAL.R), left lingual gyrus (LING.L), and right superior occipital gyrus (SOG.R). Red nodes represent target regions with significantly enhanced functional connectivity, including PreCG.R, PreCG.L, and SOG.R. Blue edges signify decreased functional connectivity between the seed and target regions, whereas red edges signify increased functional connectivity

Seed-based whole-brain FC analysis was performed using PoCG.L and SPL.L as seed regions. Compared with NCs, ABGIS_1 showed significantly decreased FC between PoCG.L and bilateral precentral gyrus (PreCG), right postcentral gyrus (PoCG.R), right middle occipital gyrus (MOG.R), and calcarine (CAL) (Table 3; Fig. 2A, Supplementary Fig. 2A). ABGIS_1 also exhibited significantly reduced FC between SPL.L and PreCG, left lingual gyrus (LING.L), right superior occipital gyrus (SOG.R), and right CAL (CAL.R) (Table 3; Fig. 2B, Supplementary Fig. 2B). Relative to ABGIS_1, ABGIS_2 demonstrated significantly increased FC between PoCG.L and right PreCG (PreCG.R) (Table 3; Fig. 2C, Supplementary Fig. 3A). ABGIS_2 also showed significantly elevated FC between SPL.L and bilateral PreCG as well as SOG.R (Table 3; Fig. 2D, Supplementary Fig. 3B). No significant alterations were found in FC between SMG.L and other regions across the whole brain, and no significant differences in FC were observed between ABGIS_2 and NCs.

**Table 3:** Brain regions with altered functional connectivity

Seed	Brain region	Cluster size	Peak MNI coordinates			T-value
			x	y	z	
ABGIS_1 < NCs						
PoCG.L	PreCG.R	118	54	6	33	4.52
	PreCG.L	66	-60	3	27	4.74
	PoCG.R	2258	27	-6	54	5.98
	MOG.R	396	30	-81	39	5.89
	CAL CAL.R CAL.L	1335 359 254	24	-63	21	5.02
SPL.L	PreCG PreCG.R PreCG.L	1891 259 214	-39	-36	63	5.30
	LING.L	161	-15	-57	-3	4.28
	SOG.R	91	30	-84	36	4.42
	CAL.R	212	21	-63	18	5.58
ABGIS_2 > ABGIS_1						
PoCG.L	PreCG.R	60	60	18	18	4.80
SPL.L	PreCG.R	327	57	3	36	5.28
	PreCG.L	65	-63	0	24	5.96
	SOG.R	81	24	-66	39	4.43

Note: Atlas parcellation: Anatomical Automatic Labeling (AAL) atlas. Voxel-level threshold: uncorrected *P* < 0.001; cluster-level threshold: Family-wise error (FWE)-corrected *P* < 0.05 (two-tailed). ABGIS_1, patients with acute basal ganglia ischemic stroke at baseline; ABGIS_2, patients with acute basal ganglia ischemic stroke at follow-up; NCs, normal controls. PoCG.L, left postcentral gyrus; SPL.L, left superior parietal lobule; PreCG, precentral gyrus; PreCG.R, right precentral gyrus; PreCG.L, left precentral gyrus; PoCG.R, right postcentral gyrus; MOG.R, right middle occipital gyrus; CAL, calcarine; CAL.R, right calcarine; CAL.L, left calcarine; LING.L, left lingual gyrus; SOG.R, right superior occipital gyrus

### Correlation analysis

**Fig. 3:**
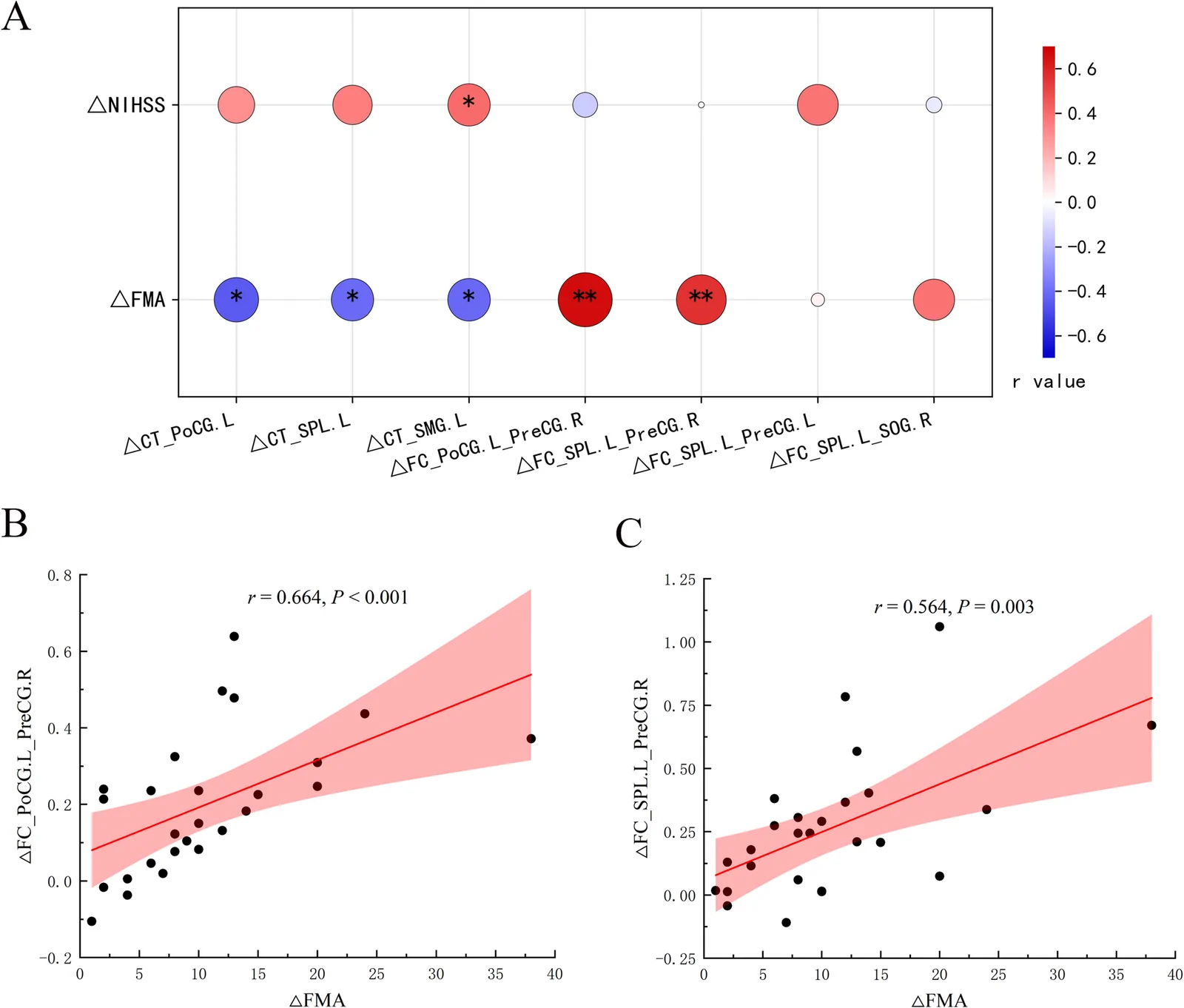
Correlations between △CT/△FC and △NIHSS/△FMA. (**A**) Correlation matrix bubble plot showing correlation coefficients (r values) between △CT/△FC and △NIHSS/△FMA. Bubble color indicates the direction and magnitude of correlation (red: positive; blue: negative), and bubble size reflects significance (*P < 0.05; **P < 0.05/14, with Bonferroni correction). (**B**) Scatter plot showing the correlation between △FC from the left postcentral gyrus to the right precentral gyrus (△FC_PoCG.L_PreCG.R) and △FMA (*r* = 0.664; *P* < 0.001). (**C**) Scatter plot showing the correlation between △FC from the left superior parietal lobule to the right precentral gyrus (△FC_SPL.L_PreCG.R) and △FMA (*r* = 0.564; *P* = 0.003). These delta values were calculated as follow-up (ABGIS_2) values minus baseline (ABGIS_1) values of patients

Figure 3A shows the correlations between △NIHSS/△FMA and △CT/△FC. The △FC between PoCG.L and PreCG.R was positively correlated with △FMA (*r* = 0.664, *P* < 0.001; Fig. 3B), and the △FC between SPL.L and PreCG.R was also positively correlated with △FMA (*r* = 0.564, *P* = 0.003; Fig. 3C).

### Classification performance and feature interpretation

**Fig. 4:**
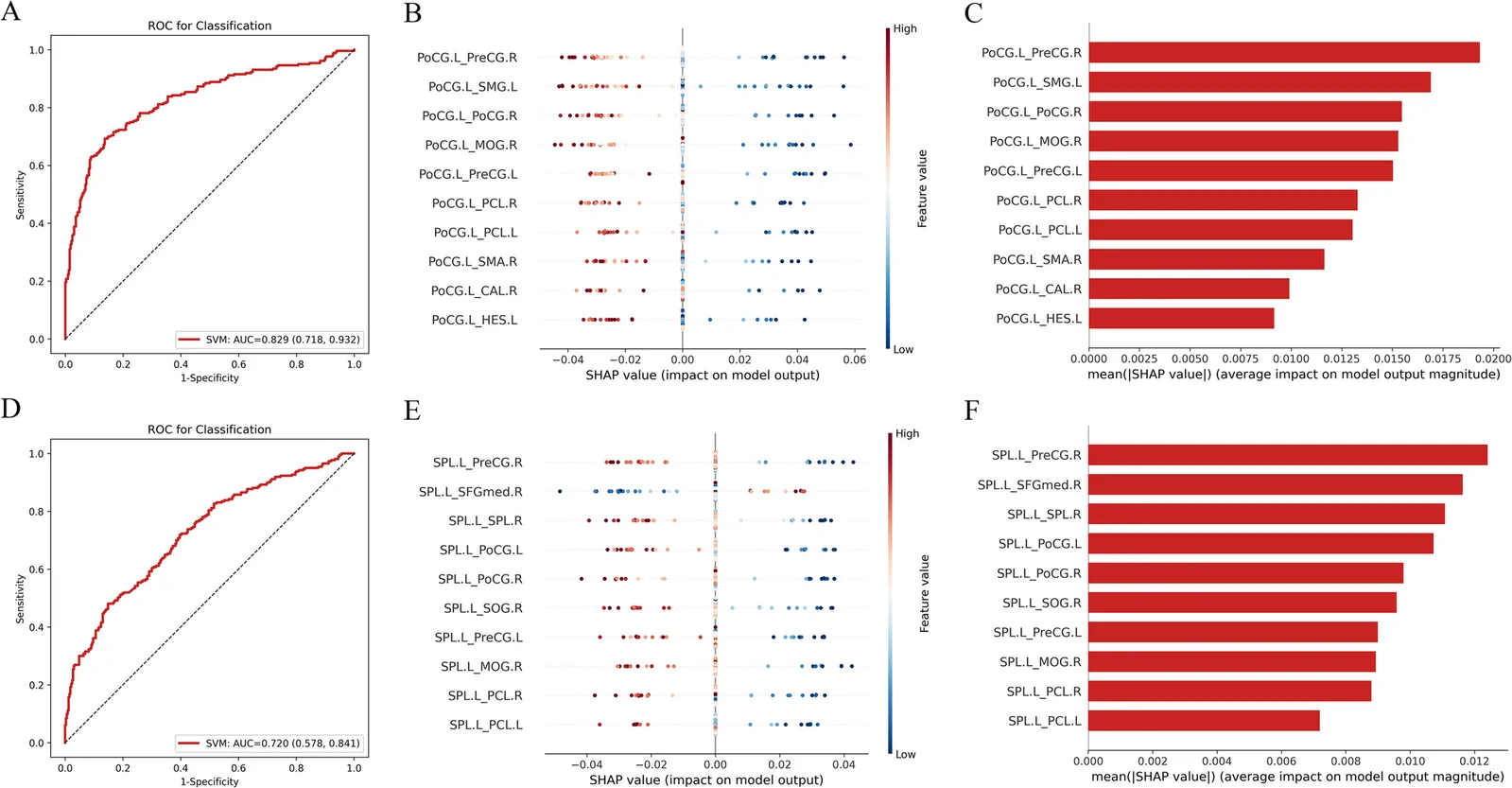
Classification performance and SHAP-based interpretation of ML models discriminating ABGIS_1 from NCs. (**A**-**C**) Results of the SVM classifier built upon the FC1 phenotype. (**D**-**F**) Results of the SVM classifier built upon the FC2 phenotype. (**A**, **D**) ROC curves. (**B**, **E**) SHAP summary plots showing the distributions of SHAP values for the top-10 predictive FC features. (**C**, **F**) Horizontal bar plots of the top-10 predictive FC features ranked in descending order by mean absolute SHAP values. For panels (**B**, **E**), red indicates higher feature values, and blue indicates lower feature values. For panels (**B**-**F**), each FC feature is labelled as region1_region2, representing the FC between region1 and region2

The whole-brain FC seeded at PoCG.L (FC1 phenotype) and SPL.L (FC2 phenotype) served as two independent feature sets for separate SVM models. The model built on the FC1 phenotype exhibited relatively higher discriminative performance than that built on the FC2 phenotype (Fig. 4A and D). When trained on the FC1 and FC2 feature sets, the SVM models yielded an AUC of 0.829 (accuracy = 0.780, sensitivity = 0.704, specificity = 0.839) and an AUC of 0.720 (accuracy = 0.661, sensitivity = 0.535, specificity = 0.761), respectively (Table 4). Among all FC features, FC between each seed region and PreCG.R showed the largest mean absolute SHAP values in their corresponding models (Fig. 4B-C and E-F; Supplementary Tables 2–3), suggesting that these connections contributed most to model predictions.

**Table 4:** Classification performance of the SVM model in discriminating ABGIS_1 from NCs

Feature set	AUC (95% CI)	Accuracy	Sensitivity	Specificity
FC1	0.829 (0.718, 0.932)	0.780	0.704	0.839
FC2	0.720 (0.578, 0.841)	0.661	0.535	0.761

Note: SVM, support vector machine; ABGIS_1, patients with acute basal ganglia ischemic stroke at baseline; NCs, normal controls; FC1, functional connectivity seeded at the left postcentral gyrus; FC2, functional connectivity seeded at the left superior parietal lobule; AUC, area under the receiver operating characteristic curve; CI, confidence interval

### Gene expression associated with FC changes

**Fig. 5:**
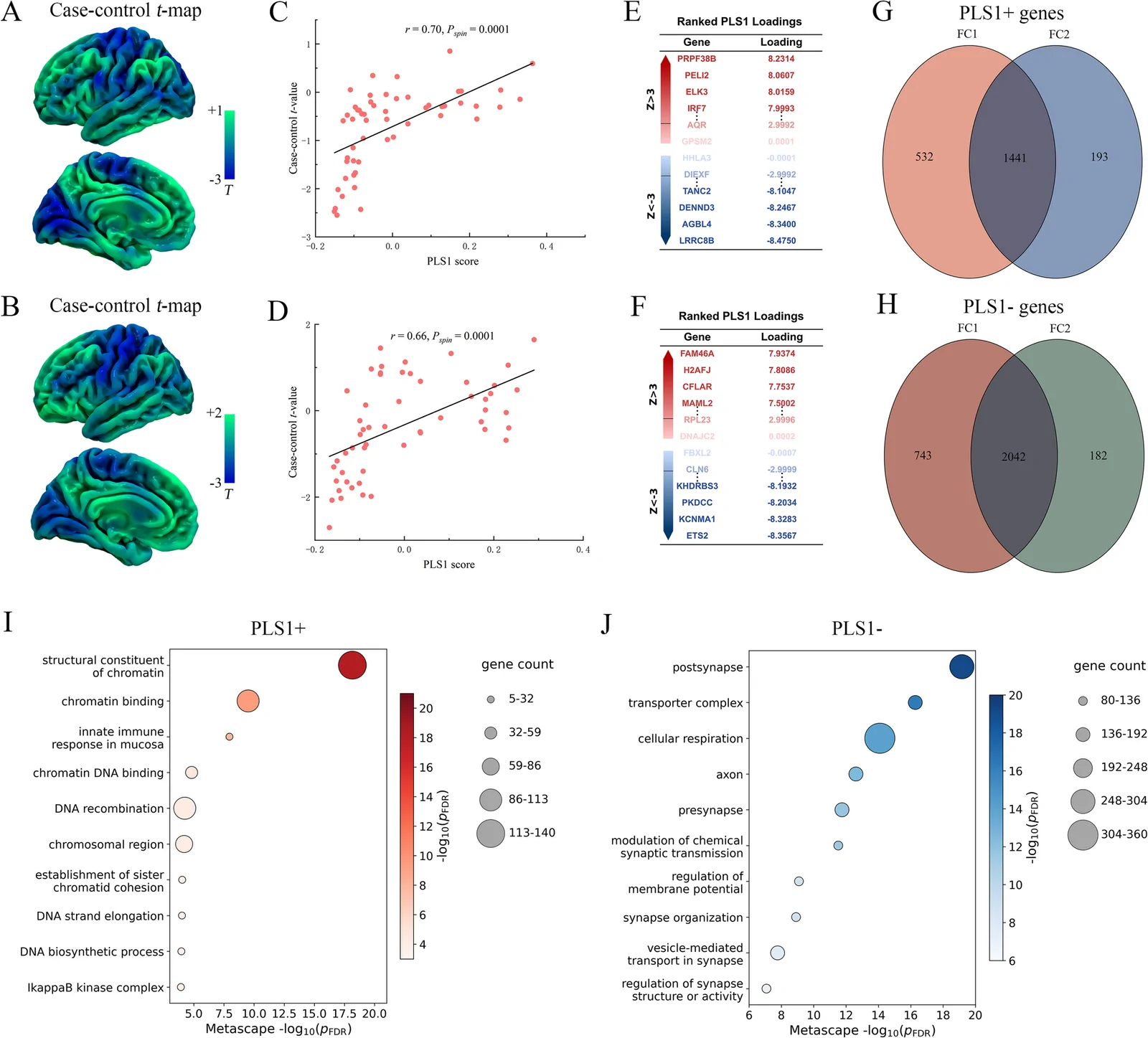
Gene expression and pathway enrichment associated with cross-sectional FC alterations. Analyses in panels (**A**, **C**, **E**) were performed on the FC1 phenotype (FC seeded at PoCG.L). Panels (**B**, **D**, **F**) correspond to analyses of the FC2 phenotype (FC seeded at SPL.L). (**A**, **B**) FC differences (case-control t-maps) in the left hemisphere. (**C**, **D**) Spatial correlation between FC differences and the PLS1 scores. (**E**, **F**) Ranked PLS1 loadings. (**G**, **H**) Venn diagrams illustrating unique and overlapping PLS1+ (**G**) and PLS1- (**H**) genes identified from separate PLS analyses of FC1 and FC2 alterations. (**I**, **J**) Bubble plots showing the top 10 significantly enriched terms for the overlapping PLS1+ (**I**) and PLS1- (**J**) gene sets

**Fig. 6:**
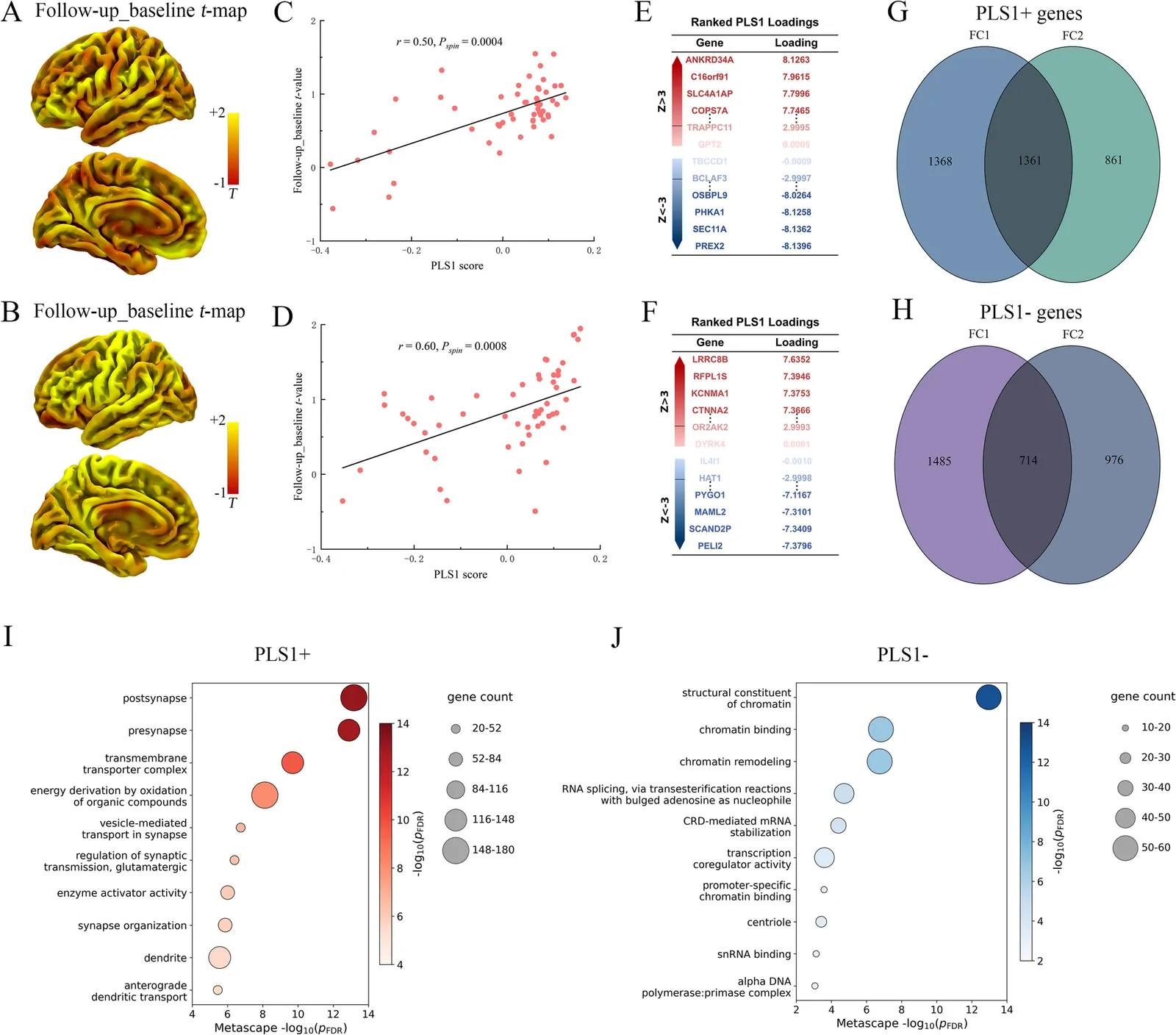
Gene expression and pathway enrichment associated with longitudinal FC alterations. Analyses in panels (**A**, **C**, **E**) were performed on the FC1 phenotype (FC seeded at PoCG.L). Panels (**B**, **D**, **F**) correspond to analyses of the FC2 phenotype (FC seeded at SPL.L). (**A**, **B**) FC differences (follow-up_baseline t-map) in the left hemisphere. (**C**, **D**) Spatial correlation between FC differences and the PLS1 scores. (**E**, **F**) Ranked PLS1 loadings. (**G**, **H**) Venn diagrams illustrating unique and overlapping PLS1+ (**G**) and PLS1- (**H**) genes identified from separate PLS analyses of FC1 and FC2 alterations. (**I**, **J**) Bubble plots showing the top 10 significantly enriched terms for the overlapping PLS1+ (**I**) and PLS1- (**J**) gene sets

We performed separate PLS regression analyses on t-maps derived from both the FC1 (Figs. 5A and 6A) and FC2 (Figs. 5B and 6B) phenotypes. For the FC1-based analyses, PLS1 explained 35.96% of the variance in the cross-sectional model (spherical permutation test, *P* = 0.0006) and PLS1 scores were significantly spatially correlated with the cross-sectional t-map (*r* = 0.70, *P*_spin_ = 0.0001; Fig. 5C). We identified 1973 PLS1 + genes and 2785 PLS1- genes (Fig. 5E). PLS1 accounted for 43.00% of the variance in the longitudinal model (spherical permutation test, *P* = 0.0002), with significant spatial association between PLS1 scores and the longitudinal t-map (*r* = 0.50, *P*_spin_ = 0.0004; Fig. 6C). This yielded 2729 PLS1 + genes and 2199 PLS1- genes (Fig. 6E). For the FC2-based analyses, PLS1 explained 30.95% of the variance in the cross-sectional model (spherical permutation test, *P* = 0.0010) and PLS1 scores were significantly spatially correlated with the cross-sectional t-map (*r* = 0.66, *P*_spin_ = 0.0001; Fig. 5D), producing 1634 PLS1 + genes and 2224 PLS1- genes (Fig. 5F). PLS1 accounted for 27.12% of the variance in the longitudinal model (spherical permutation test, *P* = 0.0041) and PLS1 scores exhibited significant spatial association with the longitudinal t-map (*r* = 0.60, *P*_spin_ = 0.0008; Fig. 6D), yielding 2222 PLS1 + genes and 1690 PLS1- genes (Fig. 6F). We identified 1441 overlapping PLS1 + and 2042 overlapping PLS1- genes for cross-sectional analyses (Fig. 5G-H), and 1361 overlapping PLS1 + and 714 overlapping PLS1- genes for longitudinal analyses (Fig. 6G-H). These shared gene sets were carried forward to functional enrichment analyses.

### Enrichment analysis of gene sets

In the cross-sectional analysis, PLS1 + genes associated with reduced FC were mainly enriched in chromatin regulation (including “structural constituent of chromatin”, “chromatin binding”, and “chromatin DNA binding”), and DNA metabolism (including “DNA recombination”, “DNA strand elongation”, “DNA biosynthetic process”, and “establishment of sister chromatid cohesion”) (Fig. 5I). In contrast, PLS1- genes were predominantly enriched in neuronal synaptic structure and synaptic signaling (including “postsynapse”, “presynapse”, “axon”, “modulation of chemical synaptic transmission”, “regulation of membrane potential”, “synapse organization”, “vesicle-mediated transport in synapse”, and “regulation of synapse structure or activity”) (Fig. 5J). In the longitudinal analysis, PLS1 + genes associated with increased FC were mainly enriched in neuronal synaptic structure and synaptic signaling (including “postsynapse”, “presynapse”, “dendrite”, “vesicle-mediated transport in synapse”, “regulation of synaptic transmission, glutamatergic”, “synapse organization”, and “anterograde dendritic transport”) (Fig. 6I). Conversely, PLS1- genes were predominantly enriched in chromatin regulation (including “structural constituent of chromatin”, “chromatin binding”, “chromatin remodeling”, “promoter-specific chromatin binding”, and “transcription coregulator activity”), as well as RNA metabolism (including “RNA splicing, via transesterification reactions with bulged adenosine as nucleophile”, “CRD-mediated mRNA stabilization”, and “snRNA binding”) (Fig. 6J).

## Discussion

In this multimodal MRI study, we found that patients with ABGIS exhibited impaired FC prior to cortical morphological changes, and showed cortical thinning in specific regions accompanied by compensatory enhancement of FC at 1-month follow-up. Correlation analysis further revealed that recovery of FC between the two thinned cortical regions (PoCG.L and SPL.L) and the contralesional PreCG was positively correlated with improvements in motor scores. SHAP analysis of both SVM models highlighted the prominent contributions of FC between each seed region (PoCG.L and SPL.L) and the contralesional PreCG to model performance. Using both FC1 and FC2 phenotypes, we generated t-maps of FC changes across the whole brain for neuroimaging-transcriptome analysis, from which we identified overlapping PLS1 + and PLS1- gene sets linked to brain functional reorganization. These gene sets associated with FC changes were predominantly enriched in neuronal synaptic structure and synaptic signaling, chromatin regulation, as well as DNA and RNA metabolism.

### Cortical morphological alterations in ABGIS

This study found no significant changes in CT among ABGIS patients in the acute stage, whereas decreased CT was observed in multiple ipsilesional parietal regions (including PoCG.L, SPL.L and SMG.L) at 1-month follow-up. Among these regions, significant CT thinning relative to NCs was restricted to PoCG.L. These findings suggest that focal basal ganglia ischemic lesions cannot induce immediate remote cortical thinning during the acute stage. Although several ipsilesional parietal regions exhibited CT thinning from the acute stage to 1-month follow-up, only PoCG.L showed cortical morphological alterations distinct from those of NCs. A longitudinal investigation into subcortical ischemic stroke demonstrated no acute structural alterations in regions remote from the primary infarct, with marked remote cortical atrophy emerging at follow-up [26]. These observations align with our findings. Such patterns of structural alterations are consistent with the theory of post-stroke diaschisis [27]. From a mechanistic perspective, accumulating evidence has clarified that damage to interregional fiber connections serves as the core intermediate link underlying such secondary cortical degeneration after subcortical infarction [28]. We therefore speculate that infarction-mediated disruption of fiber tracts connecting the basal ganglia to the ipsilateral parietal cortex triggers acute functional suppression within distal parietal regions, whereas sustained loss of afferent input ultimately leads to cortical thinning in these regions. The above three parietal subregions (PoCG.L, SPL.L and SMG.L) establish anatomical connectivity with the ipsilateral basal ganglia-thalamic axis via dense bidirectional fiber projections [2, 29, 30]. Unlike these parietal areas, the precentral gyrus receives heterogeneous subcortical afferent projections originating not only from the basal ganglia-thalamic axis but also from multiple brainstem nuclei, and this anatomical feature sustains compensatory afferent innervation following basal ganglia infarction [2]. This differential anatomical connectivity pattern may render the three parietal subregions vulnerable to cortical thinning, whereas the precentral gyrus exhibits no remarkable morphological changes at 1-month follow-up.

### Brain functional reorganization in ABGIS

Prior to detectable cortical thinning within PoCG.L/SPL.L, patients with ABGIS exhibited diminished FC linking these seed regions to sensorimotor and occipital cortices in the acute stage. Consistent with the theory of connectional diaschisis [27], such acute functional suppression in remote brain regions precedes progressive cortical thinning of the corresponding cortices. Numerous prior neuroimaging studies have consistently demonstrated extensive disruption of FC within sensorimotor and occipital cortices during the acute phase of subcortical ischemic stroke [18, 31], further corroborating our present findings that early FC impairment occurs in these specific cortical regions. Anatomically, the sensorimotor cortex consisting of the precentral and postcentral gyri functions as the core hub of the sensorimotor circuit [32, 33]. The precentral gyrus initiates voluntary motor output, whereas the postcentral gyrus refines ongoing movements via somatosensory feedback, with their synergistic interaction facilitating integrated motor control [34]. Furthermore, the occipital cortex indirectly modulates motor performance by relaying visually guided feedback signals [35]. Accordingly, impaired FC among these areas disrupts interregional information transmission, presumably leading to compromised motor function in patients with ABGIS. At 1-month follow-up, longitudinal FC analysis revealed marked adaptive functional reorganization after acute disruption of interregional connectivity, whereby impaired interregional connectivity in ABGIS patients approached normal levels. This functional remodeling consisted of elevated FC from PoCG.L to PreCG.R, as well as from SPL.L to bilateral PreCG and SOG.R. Such remodeling features resemble those reported in a prior longitudinal cohort study of stroke [18], indicating that the increased interregional connectivity may represent compensatory brain remodeling in response to early motor functional recovery after stroke.

### Functional connectivity alterations related to motor recovery and machine learning findings

Further correlation analyses were performed to clarify the clinical implications of dynamic changes in FC during the early recovery phase. The results revealed that dynamic increases in FC between PoCG.L/SPL.L and the contralesional PreCG paralleled improvements in motor function as measured by FMA. Anatomically, the precentral gyrus constitutes the primary motor cortex, which serves as the critical cortical hub mediating motor control, movement execution and skill learning [36]. This implies that the two parietal subregions exhibiting cortical thinning may facilitate motor recovery by strengthening interhemispheric signal exchange with the contralesional primary motor cortex. Consistent with our findings, an interventional study focusing on robotic-assisted therapy also verified the vital role of enhanced cross-hemispheric sensorimotor integration in boosting motor recovery after stroke [37]. Based on the framework of the interhemispheric inhibitory imbalance theory, a well-powered neuromodulation randomized controlled trial has further confirmed that enhanced physiologically beneficial interhemispheric signal exchange between ipsilesional brain regions and the contralesional precentral gyrus is critical for promoting motor recovery after stroke [38]. This suggests that compensatory strengthening of FC between the two parietal subregions and the contralesional PreCG may serve as a vital neural substrate for motor rehabilitation in ABGIS patients.

We performed SHAP analysis within the ML classification framework to identify the FC features that contributed most to model predictions. The results revealed that FC between each seed region and the contralesional PreCG exhibited the highest mean absolute SHAP values within their respective SVM classification models, highlighting the dominant contribution of these connections to model performance. Of note, our correlation analyses independently confirmed that changes in these connections were associated with motor functional recovery. These findings further corroborate the biological importance of these connections and provide preliminary clues for targeted interventions in post-stroke motor rehabilitation.

### Gene expression patterns linked to brain functional reorganization

Our integration of dynamic FC patterns with the AHBA transcriptome extends beyond conventional neuroimaging analyses, offering putative spatial biological annotations for the observed brain functional changes. Specifically, the PLS1 + genes associated with baseline FC impairment were mainly enriched in terms related to chromatin regulation and DNA metabolism, whereas the PLS1- genes showed enrichment for neuronal synaptic structure and synaptic signaling. In other words, greater FC impairment in the acute phase of ischemic stroke covaries spatially with higher expression of gene sets implicated in chromatin regulation and DNA metabolism, and with lower expression of gene sets mediating synaptic structure and synaptic signaling. Aberrant activation of chromatin regulatory pathways was identified in a murine model of acute ischemic stroke, which further contributed to endothelial injury and vascular dysfunction [39]. Mechanistic evidence synthesized in a prior review suggests that ischemia reperfusion in acute ischemic stroke can trigger homologous recombination and local DNA synthesis in postmitotic neurons [40]. Synapses represent neuronal compartments with high bioenergetic demand and are particularly vulnerable to damage during acute blood flow disturbances such as ischemic stroke [41]. Acute ischemic insult can induce suppression of synaptic function in vulnerable brain regions, characterized by disrupted presynaptic vesicle trafficking and impaired chemical synaptic transmission [42]. Taken together, our transcriptomic findings derived from the PLS analysis link these previously reported pathological processes to in vivo brain FC alterations. Notably, transcriptomic profiles in the AHBA dataset are not derived from stroke patients. Thus, this dataset cannot directly characterize the molecular mechanisms underlying FC impairments among our ABGIS participants. Nevertheless, it provides potential molecular clues for investigating brain network disturbances associated with acute stroke.

In comparison, functional enrichment of PLS1-weighted genes also provides valuable insights into transcriptomic signatures corresponding to follow-up FC recovery in patients with ABGIS. The subacute phase after ischemic stroke represents the peak window for neural plasticity, a period characterized by axonal sprouting and new synapse formation [43]. Furthermore, reparative axonal sprouting constitutes a key neuronal structural modification that mediates motor recovery after stroke in animal models [44]. Consistent with this well-established evidence, the gene set in our study positively correlated with improved FC at follow-up was predominantly enriched in neuronal synaptic structure and synaptic signaling. These findings help advance our understanding of molecular underpinnings that may support post-stroke motor recovery in ABGIS patients. In addition, the gene set negatively related to restored FC was dominantly involved in chromatin regulation and RNA metabolism. Mechanistically, observations from preclinical stroke models offer one plausible molecular interpretation for this transcriptomic signature. In mice subjected to photothrombotic stroke, neurons residing in the peri‑infarct cortex exhibited repressed RNA polymerase III‑dependent transcription during the subacute period, which constrained ribosome biogenesis and protein synthesis [45, 46]. Importantly, owing to the intrinsic limitation of the AHBA reference dataset, these mechanistic insights remain hypothetical for ABGIS patients and await further experimental validation.

Collectively, these spatial transcriptomic associations advance our mechanistic understanding of early post‑stroke brain functional reorganization. Prior human stroke neuroimaging studies have largely described macroscopic FC alterations, while animal experiments have uncovered molecular events mediating neural injury and plasticity. Our findings offer a putative spatial molecular bridge linking in vivo human brain network dysfunction to known molecular pathways observed in preclinical stroke models. Additionally, the identified transcriptomic signatures may provide preliminary clues for the development of future clinical rehabilitation strategies. These signatures highlight potential molecular candidates that could inform preclinical research on rehabilitation interventions modulating neural plasticity.

There are several limitations in this study. First, the modest sample size may both limit the power to identify subtle neuroimaging changes and compromise the generalizability of ML models. Given the limited sample size, we adopted lesion flipping to achieve unified lesion alignment. Although motor function lacks prominent hemispheric lateralization, we are still unable to fully rule out the potential confounding effects of this transformation when evaluating motor recovery in ABGIS patients. Therefore, future investigations will recruit larger cohorts and conduct subgroup analyses stratified by lesion laterality to mitigate these biases. Second, the patients enrolled in the present study predominantly presented with mild stroke, which hinders comprehensive evaluation of neuroimaging alterations across different degrees of neurological deficits in ABGIS patients. Third, acute/subacute post-stroke tissue alterations may alter GM/WM contrast and introduce bias into baseline CT quantification, and such confounding effects cannot be completely excluded in the current analysis. Fourth, the seed regions used to build classification models were identified based on group-level statistical differences in FC. Such selection of brain regions prior to model building may introduce a certain degree of selection bias, thus warranting caution when interpreting the ML results. Fifth, gene expression data are sourced from postmortem brain tissue samples from healthy human donors, whereas neuroimaging data originate from a separate stroke cohort. Although certain gene expression profiles are conserved across individuals, caution should be exercised when interpreting our results from neuroimaging-transcriptome association analyses. Finally, these association analyses using PLS cannot establish causal relationships between brain functional reorganization and gene expression profiles, and this inference warrants further validation through animal experiments.

## Conclusion

Our study delineated progressive cortical thinning and adaptive functional reorganization in the early motor recovery phase following ABGIS, and further mapped transcriptomic signatures linked to such functional reorganization. These findings extend our understanding of early motor recovery in ABGIS patients from macroscopic neuroimaging phenotypes to microscopic gene expression patterns, and offer preliminary clues for early rehabilitative interventions targeting post-stroke motor dysfunction.

## Supplementary Information

Below is the link to the electronic supplementary material.

Supplementary Material 1

## Data Availability

Raw data are available from the corresponding author on reasonable request.

## Abbreviations

ABGIS: Acute Basal Ganglia Ischemic Stroke
SBM: Surface-Based Morphometry
VBM: Voxel-Based Morphometry
CT: Cortical Thickness
GMV: Gray Matter Volume
rs-fMRI: Resting-State Functional MRI
FC: Functional Connectivity
BOLD: Blood Oxygen Level-Dependent
ML: Machine Learning
SHAP: SHapley Additive exPlanation
AHBA: Allen Human Brain Atlas
PLS: Partial Least Squares
NCs: Normal Controls
NIHSS: National Institutes of Health Stroke Scale
FMA: Fugl-Meyer Assessment
T1WI: T1-Weighted Images
TE: Echo Time
TR: Repetition Time
FA: Flip Angle
FOV: Field of View
GM: Gray Matter
WM: White Matter
CSF: Cerebrospinal Fluid
FWHM: Full-Width at Half-Maximum
SVM: Support Vector Machine
CV: Cross-Validation
AUC: Area Under the Receiver Operating Characteristic Curve
CIs: Confidence Intervals
FDR: False Discovery Rate
FD: Framewise Displacement
PoCG.L: Left Postcentral Gyrus
SPL.L: Left Superior Parietal Lobule
SMG.L: Left Supramarginal Gyrus
PreCG: Precentral Gyrus
PoCG.R: Right Postcentral Gyrus
MOG.R: Right Middle Occipital Gyrus
CAL: Calcarine
LING.L: Left Lingual Gyrus
SOG.R: Right Superior Occipital Gyrus
PreCG.R: Right Precentral Gyrus
