# Supplementary material for: Cortical thinning and functional reorganization as well as related transcriptomic signatures during early motor recovery following acute basal ganglia ischemic stroke
Source: J Neuroeng Rehabil. 2026 Nov 3;23:321. doi: 10.1186/s12984-026-02207-z (PMC13761097; doi:10.1186/s12984-026-02207-z)
Supplement: Supplementary file 1 [file 12984_2026_2207_MOESM1_ESM.docx]

**Supplementary Table 1** CT values and group comparisons for brain regions with significant differences between ABGIS_2 and NCs

| Brain region | ABGIS_2 | NCs | Mean difference [95% CI] | Hedges’ g [95% CI] | *T*-value | *P*-value |
| --- | --- | --- | --- | --- | --- | --- |
| PoCG.L | 2.00 ± 0.11 | 2.18 ± 0.12 | -0.18 [-0.24, -0.12] | -1.509 [-2.062, -0.894] | -5.830 | < 0.001 |

Note: CT, cortical thickness; ABGIS_2, patients with acute basal ganglia ischemic stroke at follow-up; NCs, normal controls; PoCG.L, left postcentral gyrus; CI, confidence interval.

**Supplementary Table 2** Stability of the top-10 predictive FC1 features ranked by mean absolute SHAP values

| FC1 features | PoCG.L_PreCG.R | PoCG.L_SMG.L | PoCG.L_PoCG.R | PoCG.L_MOG.R | PoCG.L_PreCG.L | PoCG.L_PCL.R | PoCG.L_PCL.L | PoCG.L_SMA.R | PoCG.L_CAL.R | PoCG.L_HES.L |
| --- | --- | --- | --- | --- | --- | --- | --- | --- | --- | --- |
| Replication frequency | 0.91 | 0.98 | 0.61 | 0.83 | 0.37 | 0.27 | 0.20 | 0.58 | 0.73 | 0.62 |

Note: FC1, functional connectivity seeded at the left postcentral gyrus (PoCG.L). Region1_region2 represents the functional connectivity between region1 and region2. Stability was quantified as the proportion of outer cross-validation folds in which each feature appeared in the fold-wise top-10 feature list (ranked by mean absolute SHAP values).

**Supplementary Table 3** Stability of the top-10 predictive FC2 features ranked by mean absolute SHAP values

| FC2 features | SPL.L_  PreCG.R | SPL.L_  SFGmed.R | SPL.L_  SPL.R | SPL.L_  PoCG.L | SPL.L_  PoCG.R | SPL.L_  SOG.R | SPL.L_  PreCG.L | SPL.L_  MOG.R | SPL.L_  PCL.R | SPL.L_  PCL.L |
| --- | --- | --- | --- | --- | --- | --- | --- | --- | --- | --- |
| Replication frequency | 0.77 | 0.73 | 0.41 | 0.51 | 0.53 | 0.31 | 0.56 | 0.41 | 0.21 | 0.20 |

Note: FC2, functional connectivity seeded at the left superior parietal lobule (SPL.L). Region1_region2 represents the functional connectivity between region1 and region2. Stability was quantified as the proportion of outer cross-validation folds in which each feature appeared in the fold-wise top-10 feature list (ranked by mean absolute SHAP values).

**Supplementary Fig. 1** Comparisons of clinical scale scores between ABGIS_1 and ABGIS_2. (**A**) NIHSS scores in ABGIS_1 and ABGIS_2. (**B**) FMA scores in ABGIS_1 and ABGIS_2. ^***^ *P* < 0.001.

**Supplementary Fig. 2** Cross-sectional intergroup comparisons of seed-based FC in ABGIS_1 relative to NCs. (**A**) Significant brain regions with reduced FC seeded at the left postcentral gyrus (FC1). (**B**) Significant brain regions with reduced FC seeded at the left superior parietal lobule (FC2).

**Supplementary Fig. 3** Longitudinal intergroup comparisons of seed-based FC from ABGIS_1 to ABGIS_2. (**A**) Significant brain regions with increased FC seeded at the left postcentral gyrus (FC1). (**B**) Significant brain regions with increased FC seeded at the left superior parietal lobule (FC2).
